# The association of triglycerides and total cholesterol concentrations with newly diagnosed diabetes in adults in China

**DOI:** 10.18632/oncotarget.21969

**Published:** 2017-10-19

**Authors:** Jing Cui, Jianping Sun, Wei Wang, Hualei Xin, Qing Qiao, Zulqarnain Baloch, Aiguo Ma

**Affiliations:** ^1^ Department of Nutrition and Food Hygiene, Medical College of Qingdao University, Qingdao, China; ^2^ Qingdao Municipal Center for Disease Control and Prevention, Qingdao, China; ^3^ Qingdao Institute of Preventive Medicine, Qingdao, China; ^4^ College of Veterinary Medicine, South China Agricultural University, Guangzhou, China; ^5^ Key Laboratory of Food Safety Risk Assessment, Ministry of Health, China National Center for Food Safety Risk Assessment, Beijing, China; ^6^ Department of Public Health, University of Helsinki, Helsinki, Finland

**Keywords:** triglycerides, total cholesterol, newly diagnosed, adult onset diabetes, waist circumference

## Abstract

**Background:**

It has already been suggested that high abnormal blood lipid concentration is associated with hyperglycaemia. However, no data is available about the roles of triglycerides (TG) and total cholesterol (TC) levels in diabetes. Here, for the first time we investigated the roles of TG and TC levels, gender and abdominal fat in the development of newly diagnosed diabetes in China.

**Materials and Methods:**

Two population-based cross-sectional surveys were conducted from 2006 to 2009 in Qingdao, China. Newly diagnosed diabetes was defined according to FPG and/or 2 h PG criteria. The associations between diabetes and TG, and TC levels were assessed by multi-variable logistic regression models.

**Results:**

As compared with non-diabetes, the odds ratio[(95% confidence intervals), OR(95% CI)] for diabetes corresponding to hypertriglyceridemia (HTG) were 1.54 (1.01, 2.35) in men and 2.02 (1.49, 3.10) in women for TG and accompany with Hypercholesterolemia (HTC) 2.93 (1.97, 4.37) and 2.13 (1.49, 3.05) for TC, when both were fitted simultaneously in the model adjusting for age, geographic division, marital status, school years, family history of diabetes, monthly income, systolic blood pressure (SBP), diastolic blood pressure (DBP), waist circumference (WC), high density lipoprotein cholesterol (HDL-C), alanine amino transferase (ALT) and gamma-glutamyltransferase (GGT).

**Conclusions:**

HTG in both gender, borderline high TC and HTC in men were an independent risk factor for diabetes in this Chinese population, however, HTC was mediated through abdominal fat for diabetes in women. Our findings may help to enhance the current knowledge of diabetes patho-physiology, and the associations between TG, TC level and diabetes is also clinically informative.

## INTRODUCTION

Diabetes mellitus is a group of metabolic diseases characterized by hyperglycemia over a prolonged period [1]. It is estimated that a total of415 million people live with diabetes in the world [2] and almost 193 million people have undiagnosed diabetes worldwide [3].Prevalence of diabetes is continually increasing in the world particularly in China [4, 5], mostly due to obesity, overweight, physical inactivity and unhealthy diet.

Triglycerides (TG) are the main constituents of body fat in humans [6], and irresponsible for the bidirectional transference of adipose fat and blood glucose from the liver. Total cholesterol (TC) is an important constituent (30%) of all human cell membranes, builds and maintains membranes and modulates membrane fluidity over the range of physiological processes. Different studies have demonstrated that elevated TG and TC is an independent risk factor of cardiovascular disorders [7–10]. It is generally known that diabetes was often accompanied by several potential risk factors of cardiovascular events, such as elevated TG [11, 12] and TC [13]. During last few decades, mean TG levels have increased in concert with the growing epidemic of diabetes mellitus and obesity, however, mean TC levels have decreased in America [14, 15].

In 2007, the prevalence rate of diabetes was 9.7% in China [5], which was almost two times higher than 2000–2001 (5.4%) estimates [4]. A large Chinese national survey reported in 2010 has suggested that type 2 diabetes has become a seriously public health threat in China. C-reactive protein (CRP) which is a nonspecific biomarker of acute inflammation and is normally produced in the liver. There is increasing evidence showing that elevated C-reactive protein and gamma glutamyltransferase are the potential risk factors for diabetes [16]. However, to the best our knowledge, no study has reported an association between TG and TC levels with diabetes in the world, particularly in China. Therefore, the present study was designed to investigate the associations between TG and TC concentration and the prevalence of diabetes in a Chinese population living in Qingdao, China.

## RESULTS

In this study, a total of 5012 participants were recruited, among them 2061 were men (41.1%) and 2951 were women (58.9%). The mean age of the 5012 participants was 50.7 (50.4–51.0) years with a range of 35–74 years. The mean values of FPG, 2 h PG, and HbA1c were 5.70 (5.67, 5.73), 7.34 (7.25, 7.42), and 4.46 (4.43, 4.49), respectively. The mean values of TG and TC were 1.40 (1.37, 1.43) and 5.31 (5.28, 5.34), respectively. The baseline characteristics of the participants have been shown in Table 1.

**Table 1:** Baseline characteristics of participants according to glucose

	Men		women	
	Non-diabetes	diabetes	Non-diabetes	diabetes
**Number (%)**	1793 (87.0)	268 (13.0)	2607 (88.3)	344 (11.7)
**Age (years)**	50.3 (49.8, 50.8)	55.2 (53.9, 56.5)^ǂ^	49.7 (49.3, 50.1)	56.8 (55.7, 57.9)^ǂ^
**Urban living**	587 (32.7)	107 (39.9)^*^	1436 (55.1)	178 (57.1)
**Married, *n* (%)**	1706 (96.4)	248 (93.9)	2448 (95.0)	298 (88.4)^ǂ^
**School years > 9 (%)**	673 (37.7)	89 (33.6)	836 (32.2)	60 (17.5)^ǂ^
**Current Smoking (yes, %)**	924 (51.7)	144 (54.1)	30 (1.2)	7 (2.1)
**Alcohol-drinking (yes, %)**	788 (44.1)	134 (50.2)	40 (1.5)	2 (0.6)
**Family history of diabetes (yes, %)**	245 (14.6)	62 (24.0)^ǂ^	473 (19.1)	79 (23.7)^*^
**Income (CNY/month), *n* (%)**				
**≤ 599**	595 (34.2)	95 (36.4)	1188 (47.4)	174 (52.3)^*^
**600–1999**	847 (48.7)	135 (51.7)	1179 (47.0)	155 (46.5)
**≥ 2000**	292 (17.1)	31 (11.9)	141 (5.6)	4 (1.2)
**Body mass index (kg/m2)**	25.1 (25.0, 25.3)	26.0 (25.6, 26.4)^ǂ^	25.4 (25.3, 25.5)	26.7 (26.3, 27.0)^ǂ^
**Waist circumference (cm)**	86.2 (85.8, 86.7)	89.0 (87.8, 90.2)^ǂ^	82.0 (81.7, 82.4)	85.3 (84.3, 86.3)^ǂ^
**Systolic blood pressure (mmHg)**	133.3 (132.4, 134.1)	138.7 (136.5, 140.9)^ǂ^	131.0 (130.2, 131.8)	138.5 (136.3, 140.7)^ǂ^
**Diastolic blood pressure (mmHg)**	86.1 (85.6, 86.7)	88.0 (86.5, 89.4)^*^	83.2 (82.8, 83.6)	85.9(84.7, 87.2)^ǂ^
**Fasting plasma glucose (mmol/L)**	5.48 (5.42, 5.53)	7.59 (7.44, 7.74)^ǂ^	5.46 (5.43, 5.49)	7.26 (7.17, 7.36)^ǂ^
**2 hours post-load plasma glucose (mmol/L)**	6.42 (6.30, 6.53)	12.16 (11.86, 12.46)^ǂ^	6.75 (6.67, 6.83)	12.80 (12.57, 13.03)^ǂ^
**Glycatedhaemoglobin (%)**	4.39 (4.34, 4.43)	5.12 (5.00, 5.23)^ǂ^	4.37 (4.33, 4.40)	5.06 (4.96, 5.17)^ǂ^
**High density lipoprotein cholesterol (mmol/L)**	1.63 (1.61, 1.65)	1.63 (1.57, 1.68)	1.67 (1.65, 1.68)	1.59 (1.55, 1.63)^ǂ^
**Alanine amino transferase (U/L)**	16.96 (16.22, 17.62)	21.45 (19.63, 23.27)^ǂ^	14.34 (13.87, 14.81)	16.36 (15.04, 17.69)^*^
**Gamma-glutamyltransferase (U/L)**	36.22 (32.88, 38.56)	56.09 (50.00, 62.18)^ǂ^	18.52 (17.84, 19.20)	26.47 (24.55, 28.38)^ǂ^
**Triglycerides (mmol/L)**	1.40 (1.35, 1.46)	1.93 (1.80, 2.07)ǂ	1.30 (1.25, 1.31)	1.88 (1.73, 2.03)^ǂ^
**Normal TG**	1365 (76.1)	168 (62.7)^ǂ^	2070 (79.4)	195 (56.7)^ǂ^
**Borderline high TG**	209 (11.7)	47 (17.5)	326 (12.5)	66 (19.2)
**Hypertriglyceridemia**	219 (12.2)	53 (19.8)	211 (8.1)	83 (24.1)
**Total cholesterol (mmol/L)**	5.23 (5.18, 5.27)	5.67 (5.55, 5.79)^ǂ^	5.29 (5.25, 5.32)	5.62 (5.51, 5.72)^ǂ^
**Normal TC**	925 (51.6)	85 (31.7)^ǂ^	1288 (49.4)	106 (30.8)
**Borderline high TC**	619 (34.5)	102 (38.1)	875 (33.6)	110 (32.0)
**Hypercholesterolemia**	249 (13.9)	81 (30.2)	444 (17.0)	128 (37.2)

Data are age-adjusted mean (95% confidence interval) or n (%) as indicated. ^*^P < 0.05, ^ǂ^< 0.001, non-diabetes versus diabetes within the same gender

The prevalence of newly diagnosed diabetes was 13.0% in men and 11.7% in women. Compared with non-diabetes, those individuals with newly diagnosed diabetes were older, more obese, and have higher levels of systolic blood pressure (SBP), diastolic blood pressure (DBP), ALT, TG and TC. Family history of diabetes was more common in individuals with diabetes than in those without diabetes. However, current smoking and alcohol drinking appeared to have no influence on diabetes and non-diabetes in either group.

The correlation (R^2^) for TG and TC in association with glucose and HbA1c has been given in Table 2. The levels of serum TG and TC has an independent positive correlation with FPG, 2 h PG, and HbA1c in the study population (Table 2). The OR of HTG in newly diagnosed diabetes was significantly higher (*P* < 0.001) than those with normal TG in all models. In this study, there was a significantly higher (*P* < 0.001) association among borderline high TG and diabetes in men in model 1 and 2 and in women in model 1, 2 and 3, but difference was not significant (*P* > 0.005) when both TG and TC were included in the same model (Table 2). OR value for diabetes was significantly higher (*P* < 0.001) for HTC in men and borderline high TG and HTC in women but difference was significantly higher (*P* < 0.001) when both TG and TC were included in the same model. There was also a significant interaction of WC with TG (*P* < 0.001 in men and women) and TC (*P* < 0.001 in men and women) in men and women (Table 3), and hence a stratified analysis by the WC category was further performed to investigate the association between the TG, TC and newly diagnosed diabetes. The result revealed that there was significantly higher (*P* < 0.001) association between HTG and diabetes in men, but the difference was non-significant (*P* > 0.005) after further adjustment for TG. There was no significant (*P* > 0.005) association between borderline high TG and diabetes in men of normal WC. The significant association between HTG and diabetes was evaluated in women of normal WC, and no significant association (*P* > 0.005) was found after further adjustment models. A significant association between HTC and diabetes was observed in obese women, however, not in women of normal WC (Table 3). Other results were similar to those of men and women without WC category.

**Table 2:** Standard β coefficients and R square (R^2^) for triglycerides and total cholesterol in association with fasting plasma glucose, 2 h post-load plasma glucose and glycatedhaemoglobin (%)

	Fasting plasma glucose			2 h post-load plasma glucose			Glycatedhaemoglobin		
	Standard βcoefficients	R^2^	*P*	Standard βcoefficients	R^2^	*P*	Standard βcoefficients	R^2^	*P*
**Men**									
**Triglycerides (mmol/L)**	0.124	0.101	< 0.001	0.109	0.102	< 0.001	0.119	0.135	< 0.001
**Total cholesterol (mmol/L)**	0.173	0.114	< 0.001	0.088	0.100	< 0.001	0.047	0.126	< 0.001
**Women**									
**Triglycerides (mmol/L)**	0.127	0.130	< 0.001	0.134	0.153	< 0.001	0.100	0.198	< 0.001
**Total cholesterol (mmol/L)**	0.219	0.153	< 0.001	0.113	0.148	< 0.001	0.076	0.195	< 0.001
**All**									
**Triglycerides (mmol/L)**	0.120	0.105	< 0.001	0.123	0.120	< 0.001	0.114	0.166	< 0.001
**Total cholesterol (mmol/L)**	0.193	0.123	< 0.001	0.109	0.117	< 0.001	0.068	0.159	< 0.001

Adjusted for age, geographic division, marital status, school years, family history of diabetes, monthly income, waist circumference, systolic blood pressure, diastolic blood pressure, high density lipoprotein cholesterol, alanine amino transferase and gamma-glutamyltransferase.

**Table 3:** Odds ratio (95% confidence interval) for newly diagnosed diabetes in relation to triglycerides and total cholesterol concentration

		Model 1	Model 2	Model 3	Model 4
Men					
Normal WC					
TG					
Normal TG	750	1.00	1.00	1.00	1.00
Borderline high TG	69	1.19 (0.47, 2.99)	1.19 (0.47, 3.00)	1.03 (0.40, 2.67)	0.88 (0.33, 2.31)
Hypertriglyceridemia	50	2.75 (1.26, 6.02)	2.90 (1.31, 6.43)	2.63 (1.09, 6.33)	1.94 (0.77, 4.86)
TC					
Normal TC	462	1.00	1.00	1.00	1.00
Borderline high TC	280	1.91 (1.07, 3.40)	1.92 (1.08, 3.41)	2.17 (1.18, 3.99)	2.09 (1.14, 3.87)
Hypercholesterolemia	127	4.45 (2.39, 8.30)	4.48 (2.40, 8.36)	5.23 (2.60, 10.56)	4.88 (2.40, 9.95)
Abdominal obesity					
TG					
Normal TG	783	1.00	1.00	1.00	1.00
Borderline high TG	187	1.64 (1.05, 2.56)	1.62 (1.04, 2.54)	1.54 (0.96, 2.45)	1.41 (0.88, 2.26)
Hypertriglyceridemia	222	1.90 (1.22, 2.95)	1.82 (1.17, 2.84)	1.58 (0.98, 2.57)	1.38 (0.85, 2.26)
TC					
Normal TC	548	1.00	1.00	1.00	1.00
Borderline high TC	441	1.61 (1.08, 2.40)	1.61 (1.08, 2.42)	1.57 (1.03, 2.38)	1.50 (0.99, 2.29)
Hypercholesterolemia	203	2.65 (1.68, 4.16)	2.62 (1.66, 4.13)	2.56 (1.58, 4.16)	2.40 (1.47, 3.91)
All					
TG					
Normal TG	1533	1.00	1.00	1.00	1.00
Borderline high TG	256	1.64 (1.11, 2.44)	1.56 (1.04, 2.32)	1.49 (0.98, 2.25)	1.34 (0.88, 2.04)
Hypertriglyceridemia	272	2.19 (1.51, 3.20)	2.01 (1.37, 2.96)	1.81 (1.19, 2.74)	1.52 (0.99, 2.33)
TC					
Normal TC	1010	1.00	1.00	1.00	1.00
Borderline high TC	721	1.69 (1.22, 2.34)	1.66 (1.19, 2.30)	1.65 (1.18, 2.32)	1.59 (1.13, 2.24)
Hypercholesterolemia	330	3.16 (2.20, 4.54)	3.07 (2.13, 4.43)	3.09 (2.08, 4.58)	2.89 (1.94, 4.31)
Women					
Normal WC					
TG		-			
Normal TG	1026	1.00	1.00	1.00	1.00
Borderline high TG	93	1.53 (0.67, 3.52)	1.43 (0.61, 3.31)	1.29 (0.54, 3.09)	1.34 (0.55, 3.21)
Hypertriglyceridemia	55	2.73 (1.19, 6.26)	2.63 (1.14, 6.03)	2.11 (0.87, 5.08)	2.28 (0.93, 5.59)
TC					
Normal TC	650	1.00	1.00	1.00	1.00
Borderline high TC	362	0.90 (0.49, 1.67)	0.89 (0.48, 1.65)	0.90 (0.47, 1.70)	0.85 (0.44, 1.63)
Hypercholesterolemia	162	0.87 (0.41, 1.84)	0.87 (0.41, 1.84)	0.80 (0.36, 1.77)	0.70 (0.31, 1.59)
Abdominal obesity					
TG					
Normal TG	1239	1.00	1.00	1.00	1.00
Borderline high TG	299	1.62 (1.13, 2.33)	1.55 (1.08, 2.23)	1.57 (1.08, 2.29)	1.26 (0.85, 1.87)
Hypertriglyceridemia	239	2.79 (1.94, 4.00)	2.77 (1.92, 3.98)	2.61 (1.78, 3.84)	2.01 (1.34, 3.02)
TC					
Normal TC	744	1.00	1.00	1.00	1.00
Borderline high TC	623	1.37 (0.95, 1.97)	1.37 (0.95, 1.98)	1.48 (1.01, 2.16)	1.33 (0.91, 1.96)
Hypercholesterolemia	410	2.99 (2.07, 4.30)	3.05 (2.11, 4.40)	3.43 (2.30, 5.13)	2.85 (1.88, 4.33)
All					
TG					
Normal TG	2265	1.00	1.00	1.00	1.00
Borderline high TG	392	1.68 (1.21, 2.34)	1.52 (1.09, 2.12)	1.51 (1.07, 2.13)	1.30 (0.91, 1.85)
Hypertriglyceridemia	294	3.01 (2.17, 4.19)	2.82 (2.03, 3.93)	2.59 (1.83, 3.68)	2.15 (1.49, 3.10)
TC					
Normal TC	1394	1.00	1.00	1.00	1.00
Borderline high TC	985	1.23 (0.90, 1.68)	1.21 (0.89, 1.66)	1.29 (0.93, 1.78)	1.17 (0.84, 1.62)
Hypercholesterolemia	572	2.40 (1.75, 3.30)	2.40 (1.74, 3.30)	2.58 (1.83, 3.64)	2.14 (1.49, 3.06)

Model 1: adjusted for age, geographic division, marital status, school years, family history of diabetes, monthly income, systolic blood pressure, diastolic blood pressure.

Model 2: Model 1+ waist circumference

Model 3: Model 2 + high density lipoprotein cholesterol, alanine amino transferase and gamma-glutamyltransferase.

Model 4: TG and TC fitted simultaneously into the Model 3.

## DISCUSSION

In this population-based cross-sectional study, we found that HTG and HTC were positively associated with newly diagnosed diabetes in both genders. HTC was significantly associated with newly diagnosed diabetes only in obese women, while it is a potential independent risk factor for diabetes in men of all WC categories. While, borderline high TG was also positively associated with newly diagnosed diabetes in both genders, but difference become non significance after further adjustment for TG. Borderline high TC was also positively associated with newly diagnosed diabetes in men, but not in women.

Previously reported studies had suggested that level of TG in serum was positively associated with diabetes. Moreover, it has also been shown that increased serum TG levels over time enhanced the risk of developing diabetes in various populations [17–22], these observation are consistent with our results. Many studies have reported that abdominal fatis also a primary risk factor for diabetes in the world. In the Norwegian cohort study has indicated that clinical models such as BMI,men and women aged 40–45 years; and TG is the important predictors of diabetes [23]. However, our results showed that the individual’s with HTG had a higher OR of diabetes as compared with those with normal TG in both gender, but there were non-significant differences for TG between men and women with normal WC after further adjustment. The association among impaired TG, abdominal obese and diabetes is more frequent, whereas the potential patho-physiological mechanisms are very complicated and only partially understood. Previously reported study has confirmed that abdominal obese release excessive free fatty acids, which are transported to the liver and pancreas and reduced response to insulin signaling (i.e. insulin resistance) [24]. The surplus fatty acids are restructured into triglycerides in the liver and stored into hepatocytes [25].

There is less research available on the association of TC and diabetes. In the Australian Diabetes, Obesity and Lifestyle Study and the San Antonio Family Heart Study [26], TC was strongly associated with diabetes after multivariate analysis. Results from the Multiple Risk Factor Intervention Trial study indicated that people with elevated TC has 2-flod high risk of coronary mortality in the diabetes patients as compared with controls [27, 28]. Further, there was a relationship between high total cholesterol levels and abnormalities in fasting glucose metabolism only in Diabetic patients with elevated waist circumference. In this study, we also found that the higher TC level was positively associated with pre-diabetes and diabetes in both gender, especially in women with high abdominal fat. This may be explained by the individuals with abdominal obese, abnormally in response to 2 h PG, associated with high TC level. Those subjects were characterized by high waist circumference, a poor glycemic profile and a lipid disorder (i.e. high TG level and/or high TC level). One possible interpretation of this pattern is that the excessive TC may increase dimerization of the enzyme NO synthase, which down-regulates the activity of glucokinase, thereby reducing the intra-cytoplasmic metabolism of glucose [29].

The strength of our study was the large number of subjects, the detailed analysis of the role of TG, TC level with diabetes. The application of multiple statistical models with different populations and different types of variables to evaluate the robustness of the relationship between TG, TC level and diabetes are additional strengths of this study. Third leading strength was that all newly diabetes individuals were diagnosed according to fasting plasma glucose and/or 2 h post-load plasma glucose. However, there were several limitations in this study. First, the current study was a cross-sectional study. Second, only TG and TC were performed in the current study, and the possible association of lipid disorder with newly diagnosed diabetes could not be accurately explored.

## MATERIALS AND METHODS

### Study population

**Figure 1:**
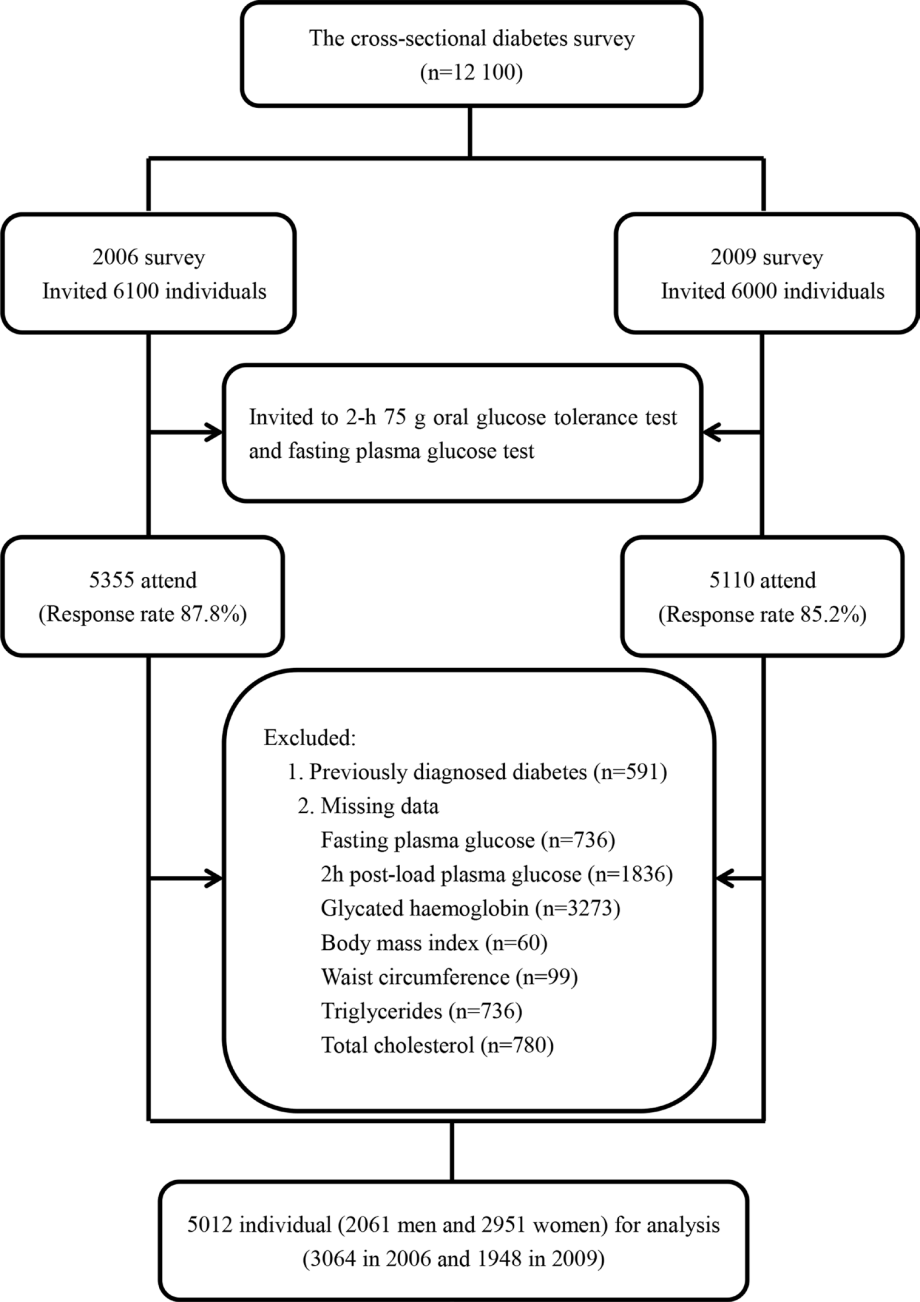
Flow chart of the participants included in the cross sectional analysis

A population-based cross-sectional diabetes survey was conducted in Qingdao in 2006 and 2009, separately. Both surveys targeted the population of three urban districts (Shinan, Shibei and Sifang) and three rural districts (Huangdao, Jiaonan and Jimo). A stratified, random cluster sampling method was used to recruit a general populationrepresentativeof 12 100 citizens (6100 in 2006 and 6000 in 2009) aged 35–74 years who have lived in Qingdao city for at least 5 years. A total of 10 465 individuals (5355 in 2006 and 5110 in 2009)were initially enrolled in this study with a response rate of 87.8% in 2006 and 85.2% in 2009, respectively (Figure 1). In this study we had used a strict inclusion criteria: 1) newly diagnosed diabetes based on fasting plasma glucose (FPG) and 2 h post-load plasma glucose (2 h PG); 2) no data missing for body mass index (BMI), waist circumference (WC), glycatedhaemoglobin (HbA1c), triglycerides, and total cholesterol level. A total of 5012 (2061 men and 2951 women) individuals were included who met the strict inclusion criteria for the analysis. Informed consent was collected from all participants. This study was approved by the local ethics committee at Qingdao Municipal Health and Family planning commission.

All eligible individuals were request to visit at the local community center, and the object of study was again explained individually. All participants were interviewed by trained interviewers using a standardized questionnaire to elicit information on socio-demographic risk factors. Marital status was classified as married/cohabiting or single/divorced/widowed). Smoking status was defined as current smokers (smoking daily regardless of the amount and type of smoking) and non-smokers (including ex-smoking, smoking now and then, not smoking at all). Alcohol-drinking status was categorized into current drinkers (drinking frequently regardless of the amount and type of alcohol) and non-drinkers (including ex-drinkers, drinking now and then, not drinking at all). Educational attainment was c7ategorized into 2 groups of school years ≤ 9y and > 9y. Personal monthly income was categorized into ≤ 599 Chinese Yuan (CNY), 600–1999 CNY, and ≥ 2000 CNY. Family history of diabetes was defined as having at least one family member (including parents, sibling and/or offspring’s) diagnosed as diabetes, without regarding the types of diabetes (Table 1).

Participant’s height and weight were measured without shoe and wearing light clothes using digital scales and a portable stadiometer, and body mass index was calculated as weight in kilograms dividing by height in meters squared (kg/m^2^). Waist circumference was measured at the middle of the rib cage and the iliac crest to the nearest 0.1 cm. Three consecutive blood pressure readings, at least 30sec apart, were taken from the upper right arm of the seated participants, and the mean value was used in subsequent data analysis.

Blood samples were collected in locally community centers, and transported in a dark box with ice to the laboratory and stored at -80 °C freezer. All blood samples were analyzed in the central laboratory of Qingdao Endocrinology and Diabetes Hospital. Standard 2 h 75g oral glucose tolerance tests (OGTT) were administrated in all participants without a prior history of diabetes. Plasma glucose was measured using glucose oxidase method. Glycatedhaemoglobin (HbA1c) was measured using an immunoturbidimetry method (Tina-qu.a A1C HIT 917 large; Roche Diagnostics). The HbA1c concentration was calculated by using the formula provided by Roche Diagnostics: [calculated HbA1c (%) = 0.81 × HbA1c (test result) + 2.39] to match the values with those found in a conventional high performance liquid chromatography (HPLC) method. Fasting serum triglycerides (TG), total cholesterol (TC) and high-density lipoprotein cholesterol (HLD-C) were measured using enzymatic methods. Serum gamma-glutamyltransferase (GGT) and alanine aminotransferase (ALT) were determined by an International Federation of Clinical Chemistry (IFCC) method.

### Classification of individuals

Individuals with previously diagnosed diabetes, who reported a history of diabetes and were under treatment with either insulin or oral anti-diabetic, were excluded from the analysis. Newly diagnosed diabetes was determined if FPG ≥ 7.0 mmol/l and/or 2 h PG ≥ 11.1 mmol/l according to the 2006World Health Organization (WHO)/International Diabetes Federation (IDF) standards [30]. According to the most recent guideline from Chinese Heart Association [31], TG and TC levels were considered normal if they were TG < 150 mg/dl and TC < 200 mg/dl, borderline high if they were 150–199 mg/dl and 200–240 mg/dl, high if they were ≥ 200 mg/dl [hypertriglyceridemia (HTG)] and ≥ 240 mg/dl [Hypercholesterolemia (HTC)]. Central obesity was defined as having waist circumference ≥ 90 cm for Chinese men and ≥ 80 cm for Chinese women [32].

### Statistical analysis

The Chi-squared test was used to compare the difference in prevalence between different glucose categories. Age-adjusted mean values and the differences of continuous variables were calculated using the general linear model in different glucose categories. The linear regression analysis was used to investigate the linear association of TG and TC with plasma glucose and HbA1c, and multivariable logistic regression analysis was used to estimate the odds ratio (OR) and 95% confidence intervals (95% CI) for the prevalence of diabetes with serum TG and TC levels in men and women. All statistical tests were two-sided; *P* values < 0.05 were considered statistically significant. Statistical analyses were performed using IBM SPSS Statistics 18.0 (SPSS, Chicago, IL).

## CONCLUSIONS

The elevated TG and TC level have highlighted a strong positive association with newly diagnosed diabetes. Additionally, borderline high TC in men and HTC in addition to being high waist circumference had a highly detrimental effect on the presence of diabetes patients in this study population. Our findings may help to enhance the current knowledge of diabetes patho-physiology, and the associations between diabetes and TG and TC level. Additionally, a future large-scale, multi-population-based studyhas been planned to further investigate the relationship between hyperglycemia and the levels of serum TC and TG.

## Abbreviations

ALT: Alanine amino transferase
BMI: Body mass index
CI: Confidence intervals
DBP: Diastolic blood pressure
FPG: Fasting plasma glucose
GGT: Gamma-glutamyltransferase
HbA1c: Glycatedhaemoglobin
HTC: Hypercholesterolemia
HTG: Hypertriglyceridemia
IDF: International Diabetes Federation
IFCC: International Federation of Clinical Chemistry
OR: Odds ratio
OGTT: 2 h 75g oral glucose tolerance tests
SBP: Systolic blood pressure
TC: TotalCholesterol
TG: Triglycerides
WC: waist circumference
WHO: World Health Organization
2 h PG: 2-hour plasma glucose
